# Changes in land-based gambling premises in London, Great Britain, 2020–2025

**DOI:** 10.1016/j.puhip.2026.100858

**Published:** 2026-09-16

**Authors:** Jenny Blythe

**Affiliations:** a Queen Mary University of London, Whitechapel, London, UK; b London School of Hygiene and Tropical Medicine, UK

## Abstract

**Objectives:**

To examine changes in the number and types of land-based gambling premises across London boroughs between December 2020 and December 2025, and to assess whether these changes were associated with borough-level deprivation.

**Study design:**

A longitudinal analysis of gambling premises across all 32 London boroughs using routinely collected regulatory data over a five-year period. Borough-level gambling availability was analysed alongside deprivation rankings derived from the 2019 Indices of Multiple Deprivation (IMD).

**Methods:**

Data on licensed gambling premises were obtained annually from the Gambling Commission register for December 2020–2025. Boroughs were ranked by IMD 2019 score, and trends in gambling premises were examined overall and by deprivation level. Comparisons were undertaken between the 25% most deprived and 25% least deprived boroughs, and a slope coefficient was used as a summary measure of the association between borough deprivation ranking and the number of gambling premises.

**Results:**

Total gambling premises in London fell from 1516 in 2020 to 1478 in 2025, a net reduction of 38 premises. However, this overall decline masked widening inequalities. The most deprived quartile of boroughs experienced a net increase of five premises, while the least deprived quartile experienced a decline of eleven premises. The positive association between borough-level deprivation and gambling premises strengthened during the study period (R^2^ increasing from 0.1082 to 0.1546).

**Conclusions:**

Despite an overall reduction in land-based gambling premises, gambling availability has become increasingly concentrated in deprived London boroughs. These findings suggest that current regulatory arrangements may be reinforcing existing health inequalities, highlighting the need for gambling policy reforms that address the unequal distribution of land-based gambling opportunities and associated harms.

## 1 Introduction

Gambling harms are increasingly recognised as a public health issue because they affect individuals, families, communities and society [1]. Harms include financial difficulties, relationship breakdown, mental and physical health problems, educational and employment impacts, and criminal activity. [1,2] Research consistently shows that gambling harms are not distributed equally; people living in more deprived areas experience greater risk. [1] Previous studies have also demonstrated that land-based gambling opportunities—including betting shops, arcades, bingo halls and casinos—are often concentrated in disadvantaged communities [[3], [4], [5], [6]].

Under the Gambling Act 2005, all land-based gambling premises in Great Britain require a premises license from a local authority. [7] The legislation contains a statutory “aim to permit” gambling unless an application conflicts with the Act's licensing objectives: preventing crime and disorder, ensuring gambling is conducted fairly and openly, and protecting children and vulnerable people from harm.

Several developments have affected the gambling sector in Great Britain during the last few years. Gambling participation has increasingly shifted online, influencing a review of legislation that is “fit for the digital age”. [8] The electronic gaming machines called Fixed Odds Betting Terminal stakes were reduced in 2019, prompting predictions of significant betting shop closures by the regulator, The Gambling Commission. [9] In addition, gambling premises faced prolonged shutdowns during the Covid-19 pandemic in 2020 and 2021, while the Gambling Act underwent a review during the same period.

London provides a particularly important case study. Although home to around 13% of Great Britain's population, it contains approximately a quarter of all land-based gambling premises. [10,11] London is also estimated to have some of the highest estimated levels of gambling-related treatment need in England. [12] Understanding how gambling premises are distributed across the capital is therefore important for both public health and policy.

This analysis examined changes in the number and type of gambling premises across London's 32 boroughs between December 2020 and December 2025 and explored whether these changes were associated with deprivation at a borough levels, where the decision-making in regard to premises licensure, is made.

## 2 Methods

Data were obtained from the Gambling Commission's online register of gambling premises [13] each December between 2020 and 2025. Premises at London borough level were classified as:

• Licensed betting offices (betting shops)
• Adult gaming centres (arcades)
• Bingo outlets
• Casinos
• Family entertainment centres

Borough-level deprivation was measured using the 2019 Index of Multiple Deprivation (IMD). [14] It reflects relative deprivation primarily using underlying administrative data from 2015 to 2016**,** and served as the official baseline for England for roughly six years until it was updated by the IMD 2025 release.

Analysis was undertaken for London overall and by premises type. In addition, the eight boroughs (25%) with the highest deprivation rankings were compared with the eight least deprived boroughs.

For each year, a scatterplot was constructed showing the total number of gambling premises in each London borough against the borough's deprivation ranking. A linear trendline was fitted using ordinary least squares regression, and the slope coefficient was extracted from the fitted equation. The slope coefficient was used as a summary measure of the association between borough deprivation ranking and the number of gambling premises.

## 3 Results

The table below summarises the extracted premises data and the calculated slope co-efficient.

**Table 1:** Premises counts and deprivation gradient (slope coefficient) by year.

Year	Total	LBO	AGC	Bingo	Casino	FEC	Deprivation slope	% change in slope from 2020
2020	1516	1313	127	38	36	2	0.108	0
2021	1495	1295	125	41	32	2	0.125	15.5
2022	1504	1296	126	49	31	2	0.126	16.4
2023	1472	1249	144	49	29	1	0.128	17.9
2024	1481	1248	148	54	30	1	0.133	22.6
2025	1478	1239	150	57	31	1	0.155	42.9

Key: LBO=Licensed Betting Offices/Betting Shops; ACG = Adult Gaming Centre; BIN=Bingo; CAS=Casino; FEC=Family Education Centre.

### 3.1 Overall trends

Between December 2020 and December 2025, the total number of gambling premises in London fell from 1516 to 1478, representing a net reduction of 38 premises. Betting shops declined by 74 venues, casinos by five and Family Entertainment Centres by one. In contrast, Adult Gaming Centres increased by 23 and bingo venues by 19.

The overall reduction, however, concealed important differences by deprivation. The eight most deprived boroughs experienced an increase in gambling premises from 425 to 430, while the eight least deprived boroughs fell from 323 to 312 premises. Consequently, the gap between the two groups widened over time. The slope of the fitted linear trendline increased from 0.1082 in December 2020 to 0.1546 in December 2025, indicating an increasingly positive relationship between deprivation rank and gambling premises counts.

### 3.2 Betting shops

Betting shops remained the most common type of gambling venue throughout the study period. Their overall number fell from 1313 in 2020 to 1239 in 2025. Nevertheless, declines were uneven.

In the 25% most deprived boroughs, betting shops decreased from 392 to 382, a reduction of only 10 venues. In the 25% least deprived boroughs, numbers fell from 289 to 267, a reduction of 22 venues. As a result, betting shops became increasingly concentrated within deprived communities despite their overall decline across London.

### 3.3 Adult gaming centres

Adult gaming centres (arcades) increased from 127 venues in 2020 to 150 in 2025. Growth occurred across London: the most deprived boroughs experienced an increase from 26 to 33 arcades, while the least deprived boroughs increased from 28 to 31.

### 3.4 Bingo outlets

Bingo premises increased substantially over the study period, rising from 38 to 57 venues across London. Growth occurred in both deprived and affluent boroughs.

In the most deprived boroughs, bingo venues increased from eight to 14. In the least deprived boroughs, they rose from four to 11. Unlike betting shops, bingo premises did not demonstrate a markedly widening deprivation gap, although overall availability increased considerably.

### 3.5 Casinos and family entertainment centres

Casino numbers declined modestly from 36 to 31 premises. Because casinos are concentrated in a small number of largely central London boroughs, deprivation-based comparisons were not undertaken. Factors such as tourism, entertainment activity and commercial property markets are likely to influence casino location patterns more strongly than deprivation.

Family entertainment centres remained uncommon and declined from two premises to one over the study period.

## 4 Discussion

The findings show that modest declines in gambling venues across London conceal a more concerning trend: land-based gambling opportunities have become increasingly concentrated in deprived communities.

Although London lost 38 gambling premises overall between 2020 and 2025, the most deprived boroughs gained venues while the least deprived boroughs experienced declines. This suggests that the association between deprivation and gambling availability strengthened during the study period.

Different premises types followed different trajectories. Betting shops declined overall but became increasingly concentrated in poorer boroughs. These findings challenge the commonly cited narrative that falling betting shop numbers inevitably represent reduced gambling exposure. The evidence suggests that reductions have occurred disproportionately in more affluent parts of the capital.

At the same time, arcades and bingo venues expanded across London, suggesting a shift in the composition of land-based gambling rather than a straightforward reduction in its availability. These findings imply that focusing solely on betting shop closures may obscure broader changes within the gambling environment.

The timing of these trends is also notable. Increases in premises within deprived communities occurred during a period when gambling venues were significantly affected by Covid-19 restrictions and while gambling legislation was under national review. These developments might have been expected to reduce physical gambling opportunities, yet the concentration of premises in deprived areas continued to increase.

Several limitations should be acknowledged. The analysis relies upon data supplied by local authorities and published by the Gambling Commission, which states that it cannot guarantee the completeness or accuracy of the register. [13] This is a concerning position for the gambling regulator, charged with enforcing the objectives of The Gambling Act that include protecting the vulnerable, to take in regard to the data that it relies upon. In addition, changes in reporting practices may also affect comparisons over time. Furthermore, the analysis was not adjusted for population size, demographic characteristics or geographical area and therefore cannot determine individual exposure to gambling opportunities.

Despite these limitations, the findings are consistent with wider evidence showing that gambling availability and gambling harms are associated with deprivation. They also raise questions about whether current licensing arrangements and legislation are effectively addressing inequalities in gambling exposure. The UK government has recently announced that it intends to remove the “aim to permit” provision from the Gambling Act, [15] but no timeframe has yet been provided. In conclusion, these findings suggest that physical gambling environments continue to contribute to health inequalities and that future gambling policy should consider not only online gambling but also the location and concentration of land-based gambling opportunities.

## Ethics statement

This analysis forms part of a doctoral thesis [16] with London School of Hygiene and Tropical Medicine (LSHTM) ethics committee approved the study in Jan 2022 (Ref 26646).

## Declaration of generative AI and AI-assisted technologies in the manuscript preparation process

During the preparation of this work, the author used Microsoft Co-Pilot for grammar and word count review in the original draft and assistance with construction of the table in the redraft. The author reviewed and edited the output as needed and takes full responsibility for the content of the published article.

## Funding

This report is independent research funded by the 10.13039/501100000272National Institute for Health and Care Research ARC North Thames. The views expressed in this publication are those of the author and not necessarily those of the National Institute for Health Research and Care or the Department of Health and Social Care.

## Declaration of competing interest

The author declares no conflicts of interest.
